# Impact of Brachyury on epithelial-mesenchymal transitions and chemosensitivity in non-small cell lung cancer

**DOI:** 10.3892/mmr.2015.3348

**Published:** 2015-02-13

**Authors:** KE XU, BIN LIU, YONGYU LIU

**Affiliations:** 1 Department of Thoracic Surgery, Liaoning Cancer Hospital and Institute, Shenyang, Liaoning 110042, P.R. China; 2 Department of Medical Oncology, Liaoning Cancer Hospital and Institute, Shenyang, Liaoning 110042, P.R. China

**Keywords:** chemotherapy, cisplatin, Brachyury, non-small cell lung cancer, epithelial-mesenchymal transition

## Abstract

The objective of the current study was to investigate the impact of Brachyury on epithelial-mesenchymal transitions and chemosensitivity in non-small cell lung cancer (NSCLC). In 115 archived NSCLC tissue samples, the expression of Brachyury was observed to be significantly higher than that in adjacent normal lung tissues. In addition, the current study demonstrated that the expression of Brachyury is closely associated with TNM staging, lymph node metastasis and the prognosis of NSCLC, although not with patient age, gender or tumor differentiation. Brachyury expression is also accompanied by the downregulation of E-cadherin and the upregulation of N-cadherin. Brachyury may promote lung cancer through induction of epithelial-mesenchymal transition, which leads to metastasis and consequent poor prognosis in patients with lung cancer. Furthermore, the present study observed that interfering with Brachyury increases the sensitivity of cells to chemotherapeutic treatment with cisplatin. These results, in combination with those of additional studies, suggest that Brachyury may be used as a novel target for the prevention and treatment of lung cancer.

## Introduction

Epithelial-mesenchymal transition (EMT) was initially described by developmental biologists, who indicated specific morphological and phenotypic alterations in epithelial cells during embryonic development (1). During this process, epithelial cells lose their polarity and become similar in morphology to fibroblasts, with reduced adhesion to the surrounding cells and matrix, thus acquiring an enhanced migratory ability (2).

It is currently hypothesized that EMT is accompanied by alterations in the expression of specific epithelial and mesenchymal markers. Epithelial cell markers such as E-cadherin, desmoplakin, tight junction protein, keratin, α-catenin, β-catenin and γ-catenin are downregulated in EMT, whereas the expression of mesenchymal tissue markers such as N-cadherin, α-smooth muscle actin, vimentin and fibronectin protein is increased (3). Furthermore, other EMT-related proteases, cytokines and transcription factors, including matrix metalloproteinase-2/9, transforming cell growth factor, fibroblast growth factor, Snail, Slug and Twist, are also upregulated (4). In addition to its role in embryonic development, previous studies have indicated that EMT is closely associated with tumor progression and resistance to chemotherapy (5). This has resulted in an increased focus on EMT by academics, clinicians and pharmaceutical researchers.

A large body of evidence indicates that the transcription factor Brachyury, which is a member of the T-box family, serves a key function during the process of EMT (5–7). T-box family members contain the highly-conserved DNA-binding domain, known as the T-box domain (8). As is the case with the other family members, Brachyury is among the proteins that are conserved during the differentiation process of the mesoderm (9–12). Previous studies have suggested that Brachyury is vital in the formation of the notochord (9). *In vitro* experiments have also indicated that Brachyury may induce mesenchymal differentiation in the embryonic stem cells of the rhesus monkey (9,11,12). A previous study demonstrated high expression levels of Brachyury in a number of types of human cancer (13), which suggests that Brachyury is important in the process of tumorigenesis, and may therefore be a novel therapeutic target in human cancer.

In the current study, the expression levels of Brachyury in normal human lung tissues and in tumor samples from patients with non-small cell lung cancer (NSCLC) were examined. The associations between Brachyury and various clinicopathological factors were analyzed in 115 NSCLC samples. The impact of Brachyury on the proliferative and invasive capacities of lung cancer cells, in addition to NSCLC cell chemosensitivity, was also investigated.

It was hypothesized that Brachyury is involved in the induction of EMT, and that via this induction, upregulation of Brachyury in NSCLC is able to exacerbate tumor malignancy.

## Materials and methods

### Patients and specimens

Ethical approval for the current study was obtained from the Ethics committee of the Liaoning Cancer Hospital and Institute (Shenyang, China). Primary tumor specimens were obtained from 115 patients diagnosed with NSCLC, who underwent complete tumor resection in the Liaoning Cancer Hospital and Institute between January and December 2007. Control samples were taken from adjacent non-cancerous normal lung tissues of the same patients. Written informed consent was obtained from each patient or their family. All 115 patients had complete follow-up records and received no radiotherapy or chemotherapy prior to surgery. The 115 patients with NSCLC comprised 80 males and 35 females, with a median age of 67.3 years (range, 47–86 years). The pathological TNM (pTNM) staging system of the Union for International Cancer Control (seventh edition) (14) was used to classify specimens as: Stage I (n=40), stage II (n=33) and stage III-IV (n=42). According to the classification of lung cancer by the World Health Organization classification guidelines, 70 cases were categorized as squamous cell carcinoma and 45 cases as adenocarcinoma.

### Immunohistochemistry

Surgically excised specimens were fixed with 10% neutral formalin (Fuzhou Maixin Biotechnology Development Co., Fuzhou, China), embedded in paraffin (Leica, Wetzlar, Germany), and cut into 4-*μ*m sections. Immunostaining was conducted using the avidin-biotin-peroxidase complex method (Ultrasensitive™; Fuzhou Maixin Biotechnology Development Co.). The expression levels of Brachyury, E-cadherin and N-cadherin were measured in 115 NSCLC specimens, using the corresponding antibodies (goat anti-human polyclonal anti-Brachyury, 1:100, sc-17745; mouse anti-human monoclonal anti-E-cadherin, 1:100, sc-8426; and goat anti-human polyclonal anti-N-cadherin, 1:100, sc-31030; all from Santa Cruz Biotechnology, Inc., Santa Cruz, CA, USA), and the correlation between the expression of these markers and clinicopathological factors was analyzed. A total of 400 tumor cells were counted and the percentage of cells with positive staining was calculated. The proportion of cells exhibiting Brachyury expression was categorized as follows: 0= absent; 1=1–25%; 2=26–50%; 3=51–75%; 4=≥76%. The staining intensity was categorized as follows: 0= negative; 1= weak; 2= moderate; 3= strong. The proportion and intensity scores were then multiplied in order to obtain a total score. To obtain final statistical results, scores <2 were considered to be negative, while scores ≥2 were considered to be positive.

### Cell culture and transfection

The A549 human lung adenocarcinoma cell line was purchased from the American Type Culture Collection (Manassas, VA, USA) cultured in RPMI 1640 medium (Invitrogen Life Technologies, Carlsbad, CA, USA) containing 10% fetal bovine serum (FBS; Invitrogen Life Technologies). Small interfering RNAs (siRNAs) for Brachyury, in addition to non-targeting siRNA, were purchased from Santa Cruz Biotechnology, Inc., and consisted of pools of three to five siRNAs to avoid off-target effects. Brachyury siRNA was transfected into the A549 cell line using Lipofectamine^®^ 2000 (Invitrogen Life Technologies).

### MTT assay

Cells were plated in four 96-well plates in RPMI 1640 medium containing 10% FBS at a density of ~1×10^5^/ml cells per well. Each plate was divided into three groups: Blank, control siRNA and Brachyury siRNA. For quantification of cell viability, 20 *μ*l 5 mg/ml MTT (Sigma-Aldrich, St. Louis, MO, USA) was added to each well at 24, 48, 72 and 96 h post-transfection. Following 4 h incubation, the media was removed from each well and the resultant MTT formazan was solubilized in 150 *μ*l dimethyl sulfoxide (Sigma-Aldrich). The results were quantitated spectrophotometrically (Bio-Rad 550; Bio-Rad Laboratories, Inc., Hercules, CA, USA) at a wavelength of 490 nm. The experiments were repeated three times and growth curves of the cells were plotted accordingly.

### Western blot analysis

Total proteins from cells were extracted in lysis buffer (Pierce, Rockford, IL, USA) and quantified using the Bradford method. Fifty micrograms of protein was separated by 10% sodium dodecyl sulfate polyacrylamide gel electrophoresis. Separated proteins were transferred to polyvinylidene fluoride membranes (Millipore, Billerica, MA, USA), and the membranes were incubated overnight at 4°C with goat anti-human polyclonal anti-Brachyury (1:100; sc-17745; Santa Cruz Biotechnology). After incubation with peroxidase-coupled anti-goat immunoglobulins (Santa Cruz Biotechnology) at 37°C for 2 h, bound proteins were visual-ized by electrochemiluminescence (Pierce) and detected by BioImaging System (UVP, Upland, CA, USA).

### Matrigel invasion assay

The bottom of the Transwell^®^ chambers (Costar, Cambridge, MA, USA) was covered with Matrigel™ (BD Biosciences, San Jose, CA, USA), 1:8, diluted in RPMI 1640 medium (~50 *μ*g/chamber). For sterilization, the prepared chambers were irradiated with ultraviolet rays for 2 h. Prior to use, a small amount of serum-free medium (Invitrogen Life Technologies) was added, in order to hydrate the chambers. A total of 0.5 ml RPMI 1640 medium containing 1% FBS was added to the lower chamber, 1.5×10/ml cells were added to the upper chamber and the chambers were subsequently incubated at 37°C for 48 h. The small chamber was removed and rinsed with PBS and the cells from the upper chamber were then removed from the microporous membrane using a cotton swab. Cells were then fixed in 95% ethanol and stained with 0.5% methylene blue (Sigma-Aldrich). The microporous membranes of the lower chamber of cells was counted under an inverted microscope (×200; TE2000; Nikon, Tokyo, Japan).

### DDP toxicity analysis

A549 cells (3×10^5^ cells/well) were cultured in a 96-well plate for 18 h. Each plate was divided into three groups: Blank, control siRNA and Brachyury siRNA, according to transfection treatment. After 48 h, 1, 3 or 5 *μ*g/ml cisplatin (DDP) was added to the culture medium of each group and incubated for 24 h. The control siRNA group with DDP treatments formed the DDP treatment groups; the Brachyury siRNA group with DDP treatments formed the combination treatment groups. The endpoint viability of the A549 cells was quantified at 24 h following DDP treatment using the MTT assay, as described above. The effect of DDP alone or in combination with Brachyury-specific siRNA on the cell proliferation inhibition rate after 24 h was calculated as follows: Cell proliferation inhibition rate (%) = (1 - the OD_570_ value of the experimental group/the OD_570_ value of the control group) × 100. The experiment was repeated three times.

### Statistical analysis

SPSS version 13.0 for Windows was used for all statistical analyses (SPSS, Inc., Chicago, IL, USA). The Pearson χ^2^ test was used to examine possible correlations between Brachyury and clinicopathological factors of NSCLC. The Kaplan-Meier method was used to evaluate the prognosis of patients. P<0.05 was considered to indicate a statistically significant difference.

## Results

### Clinical significance of Brachyury expression in NSCLC

**Figure 1:**
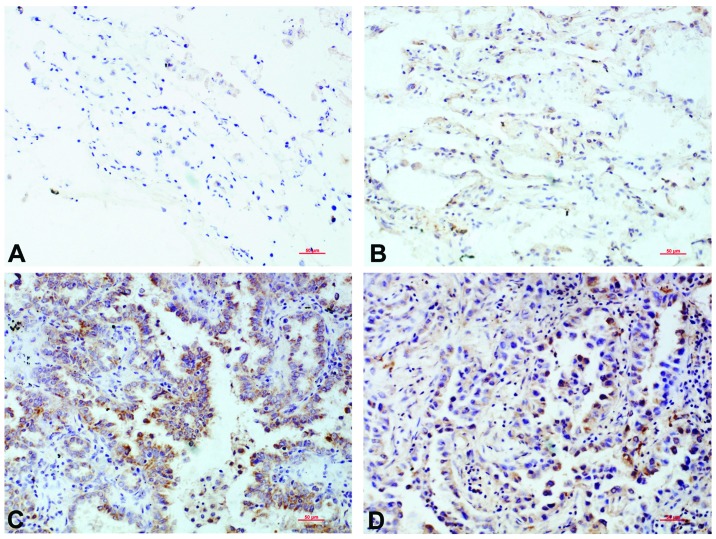
Immunostaining demonstrating the expression of Brachyury in non-small cell lung cancer tissues and matched adjacent non-cancerous lung tissues. Brachyury was negatively or weakly expressed in the cytoplasm of the matched adjacent non-cancerous normal lung tissue (A) and (B). In the lung cancer cells (C) and (D), Brachyury was predominantly located in the nucleus, with only a low level of cytoplasmic expression (magnification, ×200; red bar, 50 *μ*m).

Brachyury was observed to be negatively or weakly expressed in the cytoplasm of the matched normal lung tissues, adjacent to the cancer tissues, and was defined as negative expression according to the rating criteria (Fig. 1A and B). In the lung cancer tissues, Brachyury was predominantly located in the nucleus, with a low level of cytoplasmic expression. The proportion of cells that was positive for Brachyury expression was 40.87% (47/115), which was significantly higher than that in the normal lung tissues (0/115; P<0.001) (Fig. 1C and D).

Associations between Brachyury expression and clinicopathological factors are presented in Table I. The positive expression of Brachyury in lung adenocarcinoma was 55.56% (25/45), which was significantly higher than that in squamous cell carcinoma samples (31.43%, 22/70; P=0.01). The positive expression of Brachyury was also correlated with higher tumor stages (III+IV vs. I+II; P=0.007) and lymph node metastases (P<0.001). However, no correlation was observed between Brachyury expression and patient age, gender or tumor differentiation (P>0.05, Table I).

**Figure 2:**
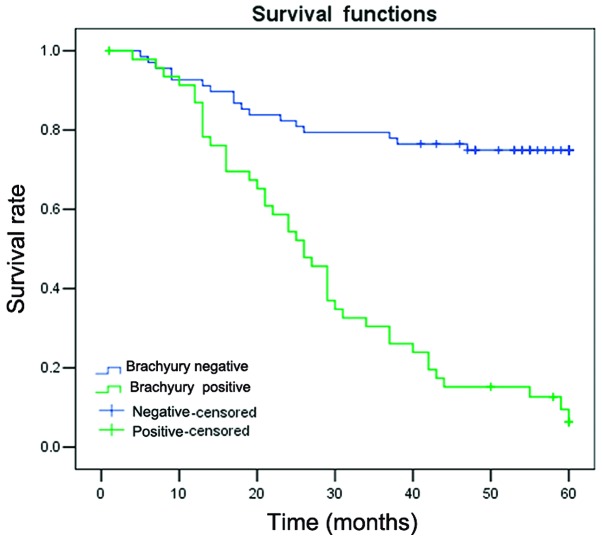
Association between Brachyury expression and survival time of patients with NSCLC. Kaplan-Meier curves for overall survival of 115 patients with NSCLC, stratified by Brachyury expression. A correlation between positive expression of Brachyury and poor overall survival was observed (P<0.001). NSCLC, non-small cell lung cancer.

Kaplan-Meier survival analysis showed that patients with Brachyury expression exhibited shorter average survival times compared with patients with negative expression of Brachyury (34.21±3.01 vs. 45.81±2.37 months; P=0.001). This suggested that Brachyury expression is associated with a poorer prognosis (Fig. 2).

### The impact of Brachyury on the expression of N-cadherin and E-cadherin in lung cancer

**Figure 3:**
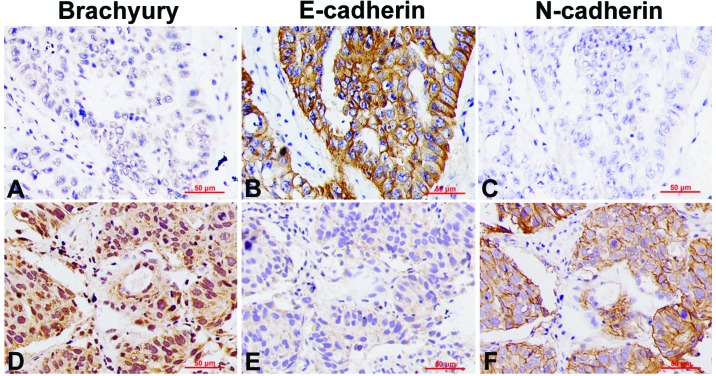
Immunohistochemical analysis of the impact of Brachyury expression on E-cadherin and N-cadherin expression in lung cancer. In Brachyury-negative (A), (B) and (C) and Brachyury-positive (D), (E) and (F) lung tissues, serial sections were stained with anti-Brachyury (A) and (D), anti-E-cadherin (B) and (E) and anti-N-cadherin (C) and (F) antibodies for immunohistochemical analysis. Magnification, ×200; red bar, 50 *μ*m.

Immunohistochemical analysis demonstrated a high expression of E-cadherin in the Brachyury-negative lung cancer tissues, with obvious staining of the cell membrane (Fig. 3A and B), whereas N-cadherin expression was either negative or low (Fig. 3C). By contrast, in the Brachyury-positive lung cancer tissues, E-cadherin expression was significantly reduced (Fig. 3D and E), while N-cadherin expression was increased, with marked membrane staining (Fig. 3F).

### Brachyury promotes lung cancer cell proliferation and invasion

**Figure 4:**
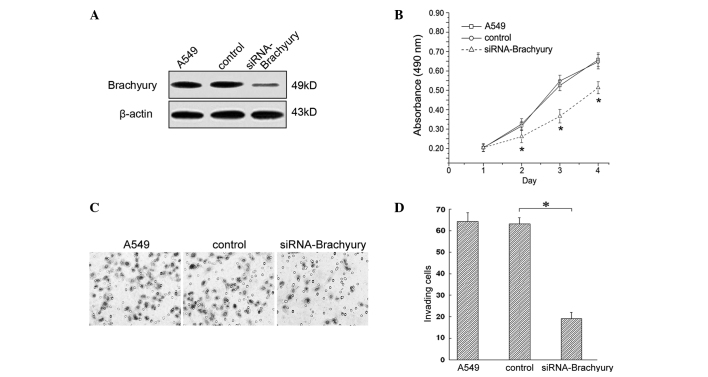
Impact of Brachyury expression on cell proliferation and invasion. (A) Expression of Brachyury following knockdown with siRNA in A549 cells. (B) Cell proliferation capacity of A549 cells was reduced following Brachyury knockdown (^*^P<0.05; n=3). (C) Invasive capacity of A549 cells was inhibited following transfection of Brachyury-specify siRNA. (D) At 48 h after Brachyury silencing, the average number of invasive cells was 19.17±2.89, which was significantly lower than that of the control group (63.47±2.93; P<0.05). Values are presented as the mean ± standard error of the mean. siRNA, small interfering RNA.

In order to study the impact of Brachyury expression on cell proliferation, an MTT assay was used. In the A549 human lung adenocarcinoma cell line, 24 h following treatment with Brachyury-specific siRNA, no significant difference in the proliferative capacity of each group was observed (P>0.05; Fig. 4A and B). However, from the second day, the lung cancer cells treated with Brachyury-specific siRNA, demonstrated a lower proliferative capacity compared with the control group (Fig. 4B, P<0.05).

A Matrigel assay was performed to study the impact of Brachyury on the invasive capacity of A549 cells. The results indicated that 48 h after Brachyury knockdown, the average number of invasive cells was 19.17±2.89, which was significantly lower than that in the control group (63.47±2.93; P<0.05; Fig. 4C and D).

### Knockdown of Brachyury increases cell sensitivity to DDP

**Figure 5:**
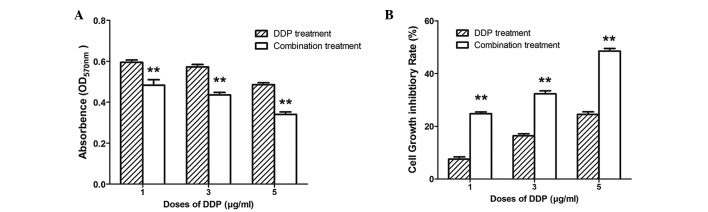
Knockdown of Brachyury increased cell sensitivity to DDP. (A) When treated with 1, 3 or 5 *μ*g/ml DDP, the cytotoxicity of the combination treatment group was higher than that of the group treated with DDP alone (^**^P<0.01). (B) Cell growth inhibition rates of the combination treatment group were significantly higher than those of the group treated with DDP alone (^**^P<0.01). DDP, cisplatin; combination treatment, DDP with brachyury-specific small interfering RNA.

DDP is a widely used chemotherapeutic drug and is frequently administered as combination chemotherapy (15). Therefore, the cell toxicity of DDP was tested in combination with silencing of Brachyury in A549 cells. In the MTT assay, the OD_570_ of cells in the control group without DDP treatment was 0.703±0.006. The OD_570_ of cells treated with 1, 3 or 5 *μ*g/ml DDP was observed to be 0.598±0.014, 0.573±0.012 and 0.490±0.013, respectively. However, when combining DDP (1, 3 and 5 *μ*g/ml) with Brachyury-specific siRNA, the OD_570_ of cells was 0.483±0.027, 0.436±0.012 and 0.334±0.018, respectively (Fig. 5A; P<0.01 between the DDP and combination groups at each corresponding concentration). This indicated that the combination of these treatment may result in a higher cytotoxicity in A549 cells than DDP treatment alone.

For cells treated with 1, 3 or 5 *μ*g/ml DDP, the cell growth inhibition rates were 7.53±0.88%, 16.40±0.73% and 24.54±0.95%, respectively. When combining DDP with Brachyury-specific siRNA, the cell growth inhibitory rates were 24.79±0.64, 32.33±1.16 and 48.54±1.00%, respectively (Fig. 5B; P<0.01 between the DDP and combination groups at each corresponding concentration). Thus, with the same concentration of DDP, there was a higher cytotoxic effect for the combined treatment group than the DDP alone group.

## Discussion

Brachyury is a T-box transcription factor, containing the highly conserved T-domain, which has the capacity to bind to DNA (5–7). Overexpression of Brachyury in human carcinoma cells is reported to induce alterations in the characteristics of EMT, including an increase in mesenchymal markers, a reduction of epithelial markers, and the upregulation of cell migration and invasion (16). However, the mechanisms underlying the involvement of Brachyury in EMT remain to be fully elucidated in human lung cancer. Therefore, the current study aimed to investigate the role of Brachyury in EMT and chemosensitivity in 115 NSCLC samples.

A previous study observed that Brachyury is highly expressed in various human tumor cell lines, but not in the majority of healthy human adult tissues (13). In accordance with this finding, the current study identified that Brachyury was negatively or weakly expressed in the cytoplasm of normal lung tissue samples, whereas it was predominantly positively expressed in the NSCLC tissues. The associations between Brachyury expression and clinicopathological factors were also investigated. In accordance with the previously proposed hypothesis that the expression of Brachyury is positively associated with tumorigenesis and malignancy (16–19), the expression of Brachyury in samples of NSCLC from patients with lymph node metastases was observed to be higher than in tumors from patients without metastases. The positive correlation between Brachyury expression and lung tumor malignancy suggests that Brachyury may be associated with lung tumor progression and makes Brachyury a potential target for lung cancer therapy.

Brachyury has widely been reported to function in the initiation and promotion of EMT (16,20). In the current study, the data obtained was consistent with that from previous studies, and suggested that in NSCLC, EMT was associated with the expression of Brachyury. E-cadherin, a cell-adhesion molecule, is a well-known suppressor of cell invasion (3,21). In lung cancer tissues with positive expression of Brachyury, the downregulation of E-cadherin may result in the initiation of EMT.

A clinical trial of a vaccine for Brachyury-positive tumors is currently underway in patients with solid tumours, as a result of further evidence supporting the hypothesis that Brachyury may be an important potential target for tumor therapy (22). In the current study, the data demonstrated that knockdown of Brachyury by specific siRNAs significantly attenuates the resistance of A549 cells to DDP treatment. Kobayashi *et al* (17) demonstrated that knockdown of Brachyury increases the sensitivity of adenoid cystic carcinoma cells to chemotherapy and radiation *in vivo*; therefore, Brachyury may be involved in the regulation of cell cycle progression, which alters the therapeutic effects of conventional cancer treatments, including chemotherapy, radiotherapy and immune therapy. Further investigation of Brachyury is required to provide more evidence for its function in cancer therapy.

In conclusion, the present study demonstrated that Brachyury expression is associated with TNM staging, lymph node metastasis and the prognosis of NSCLC. Brachyury expression is also accompanied by the downregulation of E-cadherin and the upregulation of N-cadherin. Brachyury may promote lung cancer via the induction of EMT, which leads to invasion and metastasis of NSCLC, and a consequent poor prognosis. These results, in combination with those of previous studies, support the hypothesis that Brachyury may be a novel target for the prevention and treatment of lung cancer.

## Figures and Tables

**Table I:** Summary of the correlation between Brachyury expression and clinicopathological characteristics of NSCLC.

Characteristic	Brachyury expression				
	n	Negative	Positive	χ^2^	P-value
Age (years)					
<63	46	28	18	0.096	0.757
≥63	69	40	29		
Gender					
Male	80	52	28	3.747	0.053
Female	35	16	19		
Histology					
Squamous cell carcinoma	70	48	22	6.6	0.01^*^
Adenocarcinoma	45	20	25		
Tumor differentiation					
Well to moderate	93	54	39	0.229	0.633
Low	22	14	8		
TNM stage					
I+II	73	50	23	7.25	0.007^a^
III+IV	42	18	24		
Lymph node metastasis					
Yes	61	46	15	14.25	<0.001^a^
No	54	22	32		

a P<0.05, indicate statistical significance. NSCLC, non-small cell lung cancer; n, number.
